# Afraid of the dentist? There’s an app for that: Development and usability testing of a cognitive behavior therapy-based mobile app

**DOI:** 10.1371/journal.pdig.0000690

**Published:** 2024-12-11

**Authors:** Kelly A. Daly, Kiara A. Diaz-Gutierrez, Armon Beheshtian, Richard E. Heyman, Amy M. Smith Slep, Mark S. Wolff

**Affiliations:** 1 Center for Oral Health Policy & Management, New York University College of Dentistry, New York, New York, United States of America; 2 University of Pennsylvania School of Dental Medicine, Philadelphia, Pennsylvania, United States of America; Iran University of Medical Sciences, ISLAMIC REPUBLIC OF IRAN

## Abstract

**Objectives:**

Although several brief cognitive behavior therapy (CBT)-based treatments for dental fear have proven efficacious, these interventions remain largely unavailable outside of the specialty clinics in which they were developed. Leveraging technology, we sought to increase access to treatment for individuals with dental fear through the development of a mobile application (*Dental FearLess)*.

**Materials and methods:**

To assess the resonance of our app as an avenue for dental fear treatment, we conducted a study assessing the usability, feasibility, and acceptability of the beta app. Participants with moderate to severe dental fear (*N* = 80) completed the app and reported on the perceived usability of the mobile interface (Systems Usability Scale, SUS; α = .82) and credibility of the intervention (Credibility and Expectancy Questionnaire, CEQ; α = .88). A sub-sample of participants naïve to the app (*n* = 10) completed the app during a think-aloud procedure, sharing their candid thoughts and reactions while using the app, prior to reporting on usability and credibility metrics.

**Results:**

Overall usability (*M* = 78.5, *SD* = 17.7) and credibility (*M* = 21.7, *SD* = 5.5) of the beta version of the app were good. The think-aloud data further corroborated the app’s acceptability, while highlighting several areas for user improvement (i.e., aesthetics, navigation, engagement).

**Conclusions:**

Usability and acceptability results are promising for the viability of an accessible, feasible, self-administered intervention for dental fear. Refinements made based on user feedback have produced a clinical-trial-ready mobile application. App refinement decisions, informed by user feedback, are representative of the larger literature—that is, of the ubiquitous negotiations m-health developers must make across treatment fidelity, usability, and engagement. Implications for future research are discussed.

## Author summary

Dental fear is both common and debilitating; it causes significant psychological distress and is associated with declining oral and systemic health over time. Although very effective interventions for dental fear exist, they remain widely unavailable. Self-administered mobile mental health interventions hold promise for improving access to needed psychological care for countless individuals. Although increasingly popular, research has shown that most mobile health (m-health) apps are not adapted from evidence-based psychological treatments (psychotherapies that have demonstrated their effectiveness in rigorous trials), are not vetted by mental health professionals, and are not user tested prior to release. As a team of clinical psychologists, dentists, and public health researchers, we developed a mobile app based on the most effective psychological treatment for dental fear. We then engaged in a process of user testing, which informed changes to a clinical-trial-ready version. This paper documents the process of adapting a psychotherapy treatment into mobile format, subjecting the app to user testing, and subsequent refinements to improve app quality and engagement.

## Development and usability testing of a cognitive behavior therapy-based mobile app

Over 20% of Americans who go to dentists annually suffer from moderate to severe dental fear [1], approximately double the prevalence rate of all other specific phobias combined [2]. The negative impacts of dental fear on individuals are well-documented [3–5], as are the financial costs to American society, both direct (e.g., publicly supported dental care programs) and indirect (e.g., lost productivity and absenteeism due to morbidity) [6]. Research suggests that adult dental fear rarely abates on its own [3], and the population-level prevalence of dental fear has remained stable for decades [7,8]. Avoidance of routine dental visits and preventative care due to dental fear results in oral health decline and quality of life impairment [9–11].

Brief cognitive behavioral therapy (CBT) treatments for dental fear target the fear-avoidance cycle and have demonstrated efficacy across several randomized controlled trials (RCTs) in specialty clinics [12]. Meta-analyses indicate that CBT for dental fear (CBT-DF) reduces fear and future avoidance (with large effects for self-reported fear [*d* = 1.2] and posttreatment engagement in routine oral healthcare [*d* = 1.5]) [12,13]. Despite their promise, CBT-DF treatments are largely unavailable outside of the specialty clinics in which they were developed, few mental health providers are trained to treat dental fear, and few dentists are aware that behavioral treatments for dental fear exist [14]. To address this dissemination issue, brief computerized interventions for dental fear are being developed and have shown preliminary efficacy (e.g., greater willingness to undergo dental injections [15], decreases in self-reported dental fear) [16].

We sought to increase access to care by leveraging mobile technology and developing *Dental FearLess*, a CBT-DF mobile health app accessible from a smartphone or tablet that overcomes common barriers to treatment [17]. Research (a) supports the benefits of mobile mental health apps that incorporate evidence-based treatment for anxiety and (b) demonstrates that many CBT concepts and exercises can be effectively delivered via this platform [17–19]. Mobile apps further provide flexibility [17]; in our case, patients can access the treatment when they are available and when they most need it, such as the days leading up to their dental appointment.

Although there has been a proliferation of mobile mental health apps released over the past decade [20–22], mental health experts warn that they are not created equal. Specifically, many apps are not founded on evidence-based interventions and are not designed or reviewed by practicing clinicians [17,23]. *Dental FearLess* was designed and developed by clinical psychologist practitioners and researchers (with input from a preeminent dental researcher/educator) in partnership with a technology company specializing in mobile mental health (Virtually Better, Inc).

*Dental FearLess* is a mental health-based mobile app that packages the active ingredients of CBT for dental fear (psychoeducation; cognitive, affective, and behavioral techniques; modeling; practice opportunities; and exposure) [24–29] in a self-administered accessible format. Specifically, CBT-DF psychoeducation includes (a) explanations of the fear-avoidance cycle in dental fear and the roles of cognitions, affect, and behavior in maintaining it, (b) the rationale for exposure to feared stimuli, and (c) dentistry-specific information to demystify commonly feared procedures and instruments [25,29]. Cognitive and affective techniques (standard to CBT for specific phobias) are well-validated strategies for coping with anxious reactions (e.g., recognizing and challenging distorted cognitions; diaphragmatic breathing to reduce physiological arousal) [25–29]. Behavioral strategies employ varying degrees of exposure to (a) specific feared stimuli (e.g., dental accouterment including needles for injection; common dental procedures) and (b) one’s own fear-based cognitions and emotions, and (c) directly combat avoidance [24,26,28]. As dental fear inevitably implicates a provider-patient relationship, CBT-DF also includes core behavioral strategies centered around patient-dentist communication [29]. CBT-DF necessitates modeling and patient practice of cognitive, affective, and behavioral strategies [29].

As the gold standard treatment for specific-phobias, and anxiety generally, CBT’s model of articulating fear and exposure strategies for combatting it are extremely well-validated [30,31]. Current psychological and neuro-cognitive research supports an inhibitory learning theory of exposure treatment—that is, mechanistically, exposure allows an individual to disconfirm their catastrophic beliefs regarding a feared stimulus [32] (e.g., dental injections result in facial paralysis) and incorporate new learning (e.g., dental injections maybe be uncomfortable, but are fairly safe). Modern CBT treatments for specific phobias are designed to help individuals recognize and learn from disconfirmation experiences [33]. Although CBT-DF lends itself to mobile app adaptation, adaptability is moot without adoption.

Among the most influential theories for understanding the likelihood of adoption of a new technology or innovation in public health is Roger’s Diffusion of Innovations (1962). The theory delineates barriers and facilitators that guide individuals’ perceptions of an innovation’s value via five adoption ‘drivers’ (a) relative advantage over current practice, (b) compatibility with existing attitudes or values, (c) ease of use, (d) costs in an acceptable range, and (e) observability of impacts [34]. Additionally, there is consensus in the burgeoning mobile health (m-health)-intervention field that, in addition to a strong foundation in treatments that work, a new product must be usable to have any value for its target audience [18,23]. Formal usability testing helps developers determine whether there is a market for the product and whether it matches the expectations of end-users [35]. Further, usability testing is a prerequisite for efficacy testing, as it determines necessary changes for user engagement and optimal functioning of a mobile app. The purpose of this study was to assess the usability and acceptability of *Dental FearLess (Version 1*.*0)*—a CBT-based mobile app for the treatment of dental fear—to inform a version ready for efficacy testing via a randomized controlled trial.

## Materials and Methods

### Dental fearless 1.0 app proto-type Design

**Fig 1:**
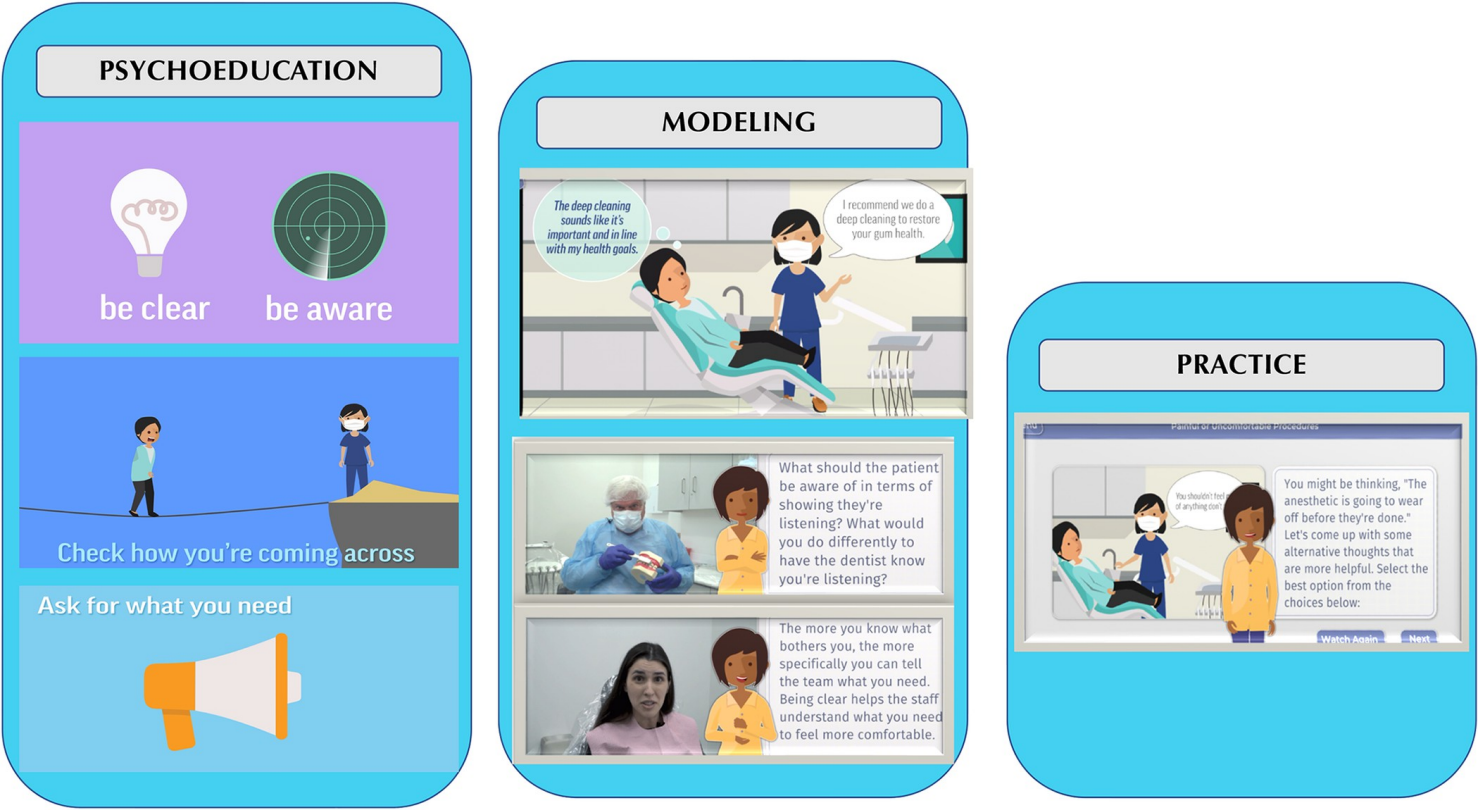
Dental FearLess Modules Setup.

To maximize accessibility and user comprehension of CBT-DF, cognitive, affective, and behavioral strategies are framed colloquially in the app as managing what you (a) think (e.g., present vs. past thinking to recognize distorted cognitions), (b) feel (e.g., progressive muscle relaxation), and (c) do (e.g., effective communication with staff) at the dentist to combat your fear. See Fig 1.

The app opens with a few questions regarding the most common dental fears [36] to determine the priority order of the specific-fear modules. Next, motivational interviewing [37] questions—aimed at increasing investment and engagement in the treatment—elicit users’ reasons for improving their dental fear.

All users then complete a comprehensive overview module providing (a) psychoeducation regarding how dental fear originates and is maintained and how to beat it (i.e., rationale for exposure), (b) information and modeling on techniques to manage thoughts, feelings and behavior in anxiety-provoking dental situations, and (c) practice on managing thoughts, feelings, and behavior. Exposure to dental equipment, personnel, and procedures occurs throughout modeling segments and user practice activities.

Users can then complete additional focal modules (presented in the order determined by an individual’s related fear rankings). App modules are: (a) General Dental Fear Symptoms and Cognitive Behavioral Strategies (the comprehensive first module), (b) A Better Dental Office: Communication and Cognition, (c) Painful and Uncomfortable Procedures, (d) Managing Bodily Symptoms, and (e) Needles and Injections. Each module follows the same format of providing relevant psychoeducation, modeling, and practice activities. Users can complete any number of modules beyond the first comprehensive one.

As they progress, users select skills or strategies for the Action Plan they intend to use at their dentist appointment. The app has patients use the evidence-based strategy of implementation intentions (simple if-then plans in the format, “If ___ happens, I will _____) to cue their strategies [38]. Also used is the “desired outcome” approach from Cognitive Behavioral Analysis System of Psychotherapy [39], whereby users assess whether their cognitions and behavior are helping them achieve their goals for dental treatment (e.g., *I would like to be able to smile more*; *I would like to be an example for my children*).

Finally, the cognitive-behavioral philosophy of the app (phrased as “Just test it”) is cognitive disconfirmation [40], whereby users plan to name their catastrophic fears and test whether they actually occur at their upcoming dental visit (e.g., “I won’t be able to tolerate sitting in the chair and will have a heart attack”). The alpha version of the app was internally tested and iterated by a team of psychologists, dental students, and research assistants/staff, some of whom had moderate-to-severe dental fear. Team members tested then-current versions of the app individually and shared their feedback during semi-monthly meetings, at which prioritized changes were determined and passed to the developer. Through this 6-month process, bugs and crashes were identified and corrected, exercises and activities were refined, and the content of some on-screen audio and visuals was changed.

### Ethics

This study was approved by the NYU Langone Health Institutional Review Board (i20-01691; i20-01994). Signed consent was obtained from all participants.

### Participants

Participants who completed the study (*N* = 80) all presented with moderate-to-severe dental fear and were 18–76 years old (*M* = 46.4; *SD =* 15.6); 73.8% identified as female (*n =* 59), 25.0% as male (*n =* 20), and 1.3% (*n =* 1) other. The sample was racially and ethnically diverse, with 40% identifying as White, 31% identifying as Black or African American, 12.5% identifying as Asian, Native Hawaiian, or Other Pacific Islander, 6.5% as multiracial, 1.25% as Native American; 8.75% declined to answer. Twenty-seven percent additionally identified as Hispanic or Latino. Participants had to complete at least the comprehensive first module of the app to continue with the study and provide feedback on usability; as such, all participants who provided data on the app metrics of interest are referred to as study “completers.” There were no obvious demographic differences between this study sample of completers (*N* = 80) and participants deemed non-completers (*N* = 9), who were withdrawn from the study, although we lacked the power to test this statistically.

### Procedure

Usability testing of the *Dental FearLess 1*.*0* App was conducted from August 2021 to August 2022 in the continental United States. Fearful dental patients were identified and referred to the study by dentists, hygienists, or front desk staff from three private practices and two university clinics as part of standard clinical practice. They were then screened by study staff. To participate, participants had to have access to a mobile device (i.e., smartphone or tablet) and endorse moderate-to-severe fear (at least 4 of 10 on a validated 0–10 single dental fear item) [41]. This single-item measure has demonstrated convergent, concurrent, discriminant, and criterion validity; [42,43,44] moreover, its brevity allowed for ease of integration into standard clinic procedures. All participants completed the informed consent procedure and a brief demographic questionnaire in REDCap (a secure web-based program for HIPAA-compliant collection, management, and storage of participant data). Each individual signing consent was offered the opportunity to either complete the app on their own time or participate in a “think aloud” session (detailed below) with a member of the study team, until the think-aloud subsample was attained (*n* = 10).

Seventy-nine participants received instructions for downloading and accessing the *Dental FearLess 1*.*0* app independently. Participants were encouraged to complete the app in one sitting during their initial use. Each module was completed in total, and modules were presented in order of fear rankings (i.e., participants could not skip ahead or click through module content without completing each segment). The app recorded usage metrics (i.e., fear areas endorsed, additional modules completed, answer selections for specific items embedded in module exercises, and time spent). Nine of these individuals downloaded and initiated the app but did not complete at least one module (spending an average of 8.25 minutes on the 60–90-minute program); these non-completers were ineligible to provide follow-up reporting, and their data (including demographic data) were excluded from this study sample.

To be deemed an app completer and continue with the study participants had to finish at least the introductory comprehensive module “General Dental Fear: Symptoms and Cognitive Behavioral Strategies,” which included psychoeducation, modeling, and practice. The general dental fear module begins with 21 minutes of animated psychoeducation video regarding (a) the etiology of dental fear, (b) associated symptoms and maintenance factors, (c) the app’s primary CBT premise and (d) affective, behavior, and cognitive coping strategies. Next, participants complete five activities focused on: (a) what the user wants to get out of the app, (b) how they currently feel about going to the dentist, (c) communication skills to try, (d) creating a plan for a different dental experience, and (e) emotion regulation strategies—progressive muscle relaxation and diaphragmatic breathing. Time spent on activities can vary substantially; the emotion regulation strategies include nine minutes of audio-visual guidance guided practice. The first module ends with two interactive modeling-and-practice exercises for (a) communication skills and (b) challenging cognitions commonly evoked in dental situations. Among completers, 28% finished the minimum (module one), whereas 72% also completed at least one additional module focused on a specific fear area(s). Users reported their app completion to the study coordinator, who verified via app tracking metrics. Following, questionnaires about usability, credibility, and drivers of adoption were made available to participants via REDCap. The drivers, informed by the diffusion of innovation theory, provide measures of individuals’ preparedness to use, and acceptance of, the mobile app. Together, usability, credibility, and drivers reflect the likelihood of using the app practices for future dental visits.

Usability scales are frequently employed in technological testing. Although they can produce global indicators of user experience—positive and negative—they do not allow developers to identify areas for modification and improvement. As such, following mobile-app development best practices, [35] we conducted think-aloud testing with a small subsample of participants (*n* = 10) (i.e., these naïve participants used the app for the first time and provided their internal monologue to a silent facilitator throughout use) via Zoom. Think-aloud testing procedures are considered the most effective way to obtain extensive and comprehensive feedback about the User Experience (UX) and User Interface (UI) of a mobile app [35,45]. Participants were given the following instructions: “We are going to ask you to “Think Aloud”, sharing your inner reactions the whole time. We want you to be completely candid; we are not evaluating you. We are using this information to evaluate our app. We’d like you to share all of your thoughts and feelings as they pop into your head, including when something does or doesn’t meet your expectations, surprises you, confuses you, delights you, or frustrates you, and why.”

After completing app testing, participants filled out questionnaires and answered interview questions about their reactions to specific features of the app (e.g., layout, style, content, resonance) and their thoughts about the intervention experience. Think-aloud sessions lasted 70–120 minutes and were recorded. This mixed methods approach is standard practice in assessing an electronic health product’s usability [45,46].

### Measures

***Systems Usability Scale*** (SUS) [47]. All participants completed a 9-item version of the SUS [48], the most widely used measure of usability. The SUS is an accessible reliable means of assessing overall usability of mobile health apps. It is brief, easily administrable, and has strong psychometric properties [49]. Each item (e.g., “There was too much inconsistency in the *Dental FearLess* app”) was rated on a 5-point Likert scale from “strongly disagree” to “strongly agree.” Specific items are reverse-coded. SUS scoring was performed as per author instructions including adjustment for administration of the nine-item version [48], producing a total score between 0 and 100. Internal consistency in this sample was good (α = .82 in this sample).

***Credibility and Expectancy Questionnaire*** (CEQ) [50,51]. The CEQ’s 3-item credibility subscale was administered following app completion. The CEQ is the only brief self-report scale explicitly designed to the assess the perceived credibility of a mental health intervention. Credibility—or how believable, resonant, and logical a psychotherapy intervention seems—predicts individuals’ motivation, engagement, and symptom improvement across treatment [51,52]. The CEQ is brief, accessible, and widely administered; the credibility subscale has strong psychometric properties [51]. Each item (e.g., “How confident would you be in recommending *Dental FearLess* to a friend?”) is scored on a 9-point Likert scale from “not at all confident” to “very confident.” Items are summed to produce a total score between 3 and 27. Internal consistency was high (α = .88).

***Drivers of Innovation***. Based on the diffusion of innovation theory [34], participants completed a 7-item scale assessing the five drivers that inform the likelihood that a new technology is adopted. The drivers are (a) relative advantage over current practice, (b) compatibility with existing attitudes or values, (c) ease of use, (d) costs in an acceptable range, and (e) observability of impacts. Each item (e.g., “The way I usually prepare for my dental visits is just as good or better at managing my dental fear as the *Dental FearLess* app”) was scored on a five-point Likert scale from “strongly disagree” to “strongly agree.” Two drivers (costs and observability) included two items that were averaged for scoring; the other three drivers were each assessed using one item. Two items were reverse-coded. The author-created scale had good internal consistency (α = .83).

***Think-aloud Data Preparation*.** All think-aloud recordings were first transcribed by Otter.ai software and then checked and corrected by staff members listening to the recording. Two trained coders read the transcripts; using thematic analysis processes [53], they identified and distilled user reactions to specific attributes of the app (e.g., positive and negative statements regarding content, appearance, and functionality) into commonly appearing categories. In cases when coders disagreed about a primary theme indicated by a user comment, they met with the first author, and all parties discussed until consensus was reached.

## Results

There were no obvious demographic differences between participants who did independent usability testing (*n* = 70) and those who did the think-aloud task (*n* = 10).

### App usage

**Fig 2:**
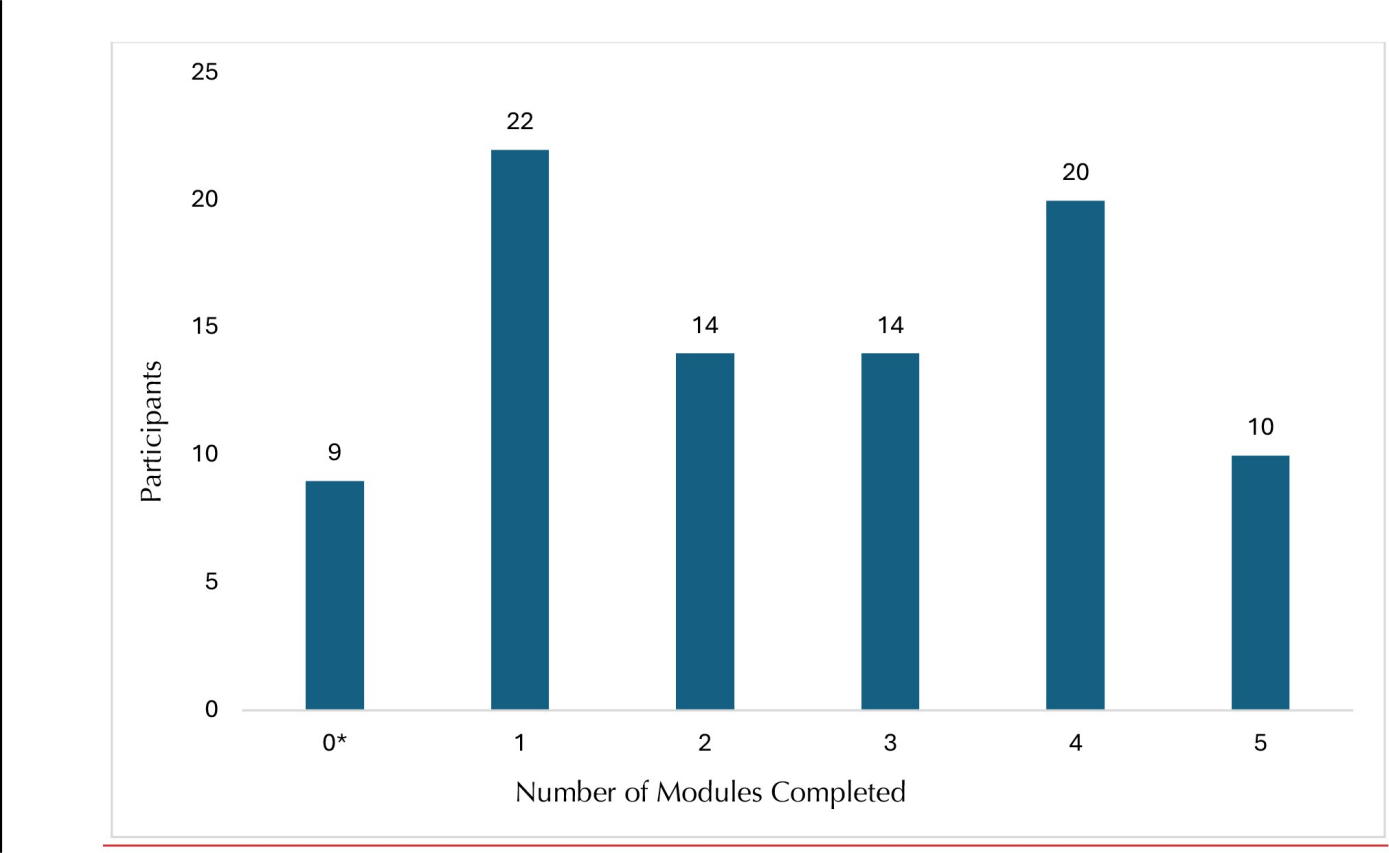
Module Completion Across Participants. *Note*. * Denotes participants who did less than the mandatory first module (0 total completed modules). were ineligible to complete follow-up questionnaires, and were withdrawn from the study.

Participants finished *M* = 2.75 (*SD* = 1.4) app modules, and spent an average of *M* = 81.8 (*SD* = 25.1) minutes on the app (time spent ranged from 43 to 154 minutes across participants). Amount of time spent accessing the app did not differ among those who completed the app independently and those who did the think-aloud (*t* (78) = .84; *p =* .40). When first opening the app, participants rated the importance of improving their dental fear on a 0–10 scale (*M* = 8.1, *SD* = 2.1), along with the aspects of the dentist they find most anxiety-provoking. See Table 1. As shown in Fig 2, total modules finished ranged from 1 (the comprehensive first module) to 5 (all four-specific fear-area modules). Table 2 indicates the topics of specific additional modules finished by completers.

**Table 1:** Aspects of the Dental Visit Assigned Highest Fear Ratings.

	Percent	*n*
Painful or uncomfortable procedures	29	23
Bodily symptoms (e.g., heart racing, difficulty breathing, sweating)	26	21
Needles and injections	24	19
Dental staff being unsympathetic or unkind	23	18
Not being in control	20	16
Feeling embarrassed or ashamed	19	15
Not knowing what the dentist is going to do	16	13
Feeling sick, queasy, or disgusted	13	10
Gagging or choking	9	7
Numbness caused by the anesthetic	9	7

*Note*. Participants could assign multiple categories the highest rating; as such, percentages do not sum to 100 and *n*s do not sum to the sample size.

**Table 2:** Specific Modules Completed by Sample Participants.

Module	Completed
General Dental Fear: Symptoms and Cognitive Behavioral Strategies *	100%
A Better Dental Office: Communication & Cognition	49%
Painful Uncomfortable Procedures	45%
Managing Bodily Sensations	30%
Needles & Injections	21%

*Note*.

*Required for inclusion in the study sample

### Usability and credibility

SUS ratings ranged from 17 to 100 (*M* = 78.6, *SD* = 17.7). The mean SUS score was significantly higher (*t* (79) = 5.34, *p* < .001, Cohen’s *d* = .60, 95% CI [.36, .83]) than the SUS cut score of 68, established as the minimum usability for m-health apps [54]. Twenty-nine percent of users (*n* = 23) rated the app at or below the cut score. The 1.0 version of the app’s average SUS score equates to a B+ (80–84^th^ percentile) rating for overall usability [55]. The subsample of think-aloud participants rated the app higher regarding usability (*M* = 89.4) than participants who accessed the app independently (*M* = 77.2; unequal variances *t* (20) = -3.4; *p* = .003). The average rating among participants who accessed independently equates to the same B+ usability score category as the overall sample mean.

Scores on the credibility (believability, logical, helpfulness of the intervention) subscale of the CEQ ranged from 3 to 27 (*M* = 21.7, *SD* = 5.5). Mean credibility was significantly higher than a “somewhat credible” score of 15 (*t* (79) = 10.9, *p* < .001; Cohen’s *d* = 1.2, 95% CI [.93, 1.5]). The think-aloud subsample rated credibility (*M* = 24.8; *SD* = 2.7) higher than those who accessed independently (*M* = 21.2; *SD* = 5.6; unequal variances *t* (22) = -3.3, *p* = .004). The mean credibility score among those who accessed independently mirrored the sample’s overall average credibility. Eighty-three percent of participants (*n* = 66) rated credibility higher than “somewhat.”

### Adoption and helpfulness

Most participants rated the *Dental FearLess 1*.*0* app favorably on the key features theorized to drive the adoption of innovations (see Table 3). Favorability ratings averaged 71% and ranged from 60% to 79% percent.

**Table 3:** Five Drivers of Innovation Adoption for Dental FearLess 1.0 m-Health App.

	Agreement		
Driver	Somewhat	Strongly	Sum
Relative Advantage (“better than the way I usually prepare”)	29%	31%	60%
Compatibility (“fit into my dental care”)	25%	49%	74%
Ease (“no struggle to participate”)	18%	48%	66%
Costs (“time worth it; overall worth it”^†^)	36%	43%	79%
Observability (“something innovative; beneficial”^†^^)^	31%	47%	78%

Note: *N* = 80

^†^ mean of two items computed for driver

### Think-aloud testing data and follow-up interviews

Individual testing sessions indicated convergence across several areas for improvement. Overall, participants liked the app animations, expressing they found them helpful for sustaining attention and increasing engagement with something that can feel unappealing (i.e., coping with dental visits). Participants had six key suggestions. First, participants requested the ability to pause and rewind video and animated content and to return to a module later. Second, participants wished there was a way to track their progress through the app, know how much content was left in any section, and be cued when an app segment was completed. Third, during the occasional instance that screens were text-only, participants expressed a desire for more animation, more color, and layouts less reflective of “high school textbooks.” Some screens also had an overwhelming amount of text that replicated what was being said on the audio, making it harder to attend to the audio. Fourth, participants believed there were too many questions about their dental fear at the beginning. Fifth, participants commented on the length of the app, with some people spending up to two hours in one sitting getting through the material (when told that it would take 60–90 minutes). Finally, participants enjoyed the app segments devoted to emotional coping techniques (evidence-based diaphragmatic breathing and progressive muscle relaxation [PMR] exercises) and wished they could jump directly to these components at any point while using the app.

*Interviews*. During the brief follow-up interview segments, participants reported finding the overall experience of using the app “normalizing” and helpful. Although participants who piloted the injection-fear module commented that it felt particularly lengthy, they also acknowledged its utility. Two talked about feeling inspired to find other ways to get more comfortable with the sight of needles. Several participants found that the communication module provided them with a sense of permission to ask questions they had previously felt embarrassed about. They also found the guidance to “ask for what you need” empowering. Several participants expressed doubt they would be capable of challenging their fear-based cognitions (past vs. present-focused thinking, assuming positive intent of dental staff) at the dental visit. Participants reiterated the helpfulness of the evidence-based emotion-coping techniques during interviews.

### App refinement

Based on participant feedback, changes were submitted to the app developer for *Dental FearLess 2*.*0*, the RCT-ready version (see Table 4). Individual progress tracking and module completion awareness were added to the app, reflecting common usability design practices.

A colored progress bar was added to the bottom of every screen, and a celebratory message is now shown after each completed module. Several additional revisions were made to the UI for improved app navigation, including user controls (e.g., pause, rewind), an always-accessible home menu with visual icons for easy-to-access emotional coping strategies (diaphragmatic breathing, progressive muscle relaxation), and the user’s appointment gameplan (i.e., strategies the user has chosen to employ at their next dental visit). We shortened the initial dental-fear stimuli assessment and reduced the amount of onscreen text accompanying coaching voiceovers. Finally, to shorten the app completion time, we streamlined how participants progress through the commonly used injection module. In addition, app navigation was added to allow user access to all other modules, if desired (e.g., rather than having to progress them in in a hierarchical order after introductory module and highest rated fear module (e.g., painful and uncomfortable procedures)).

**Table 4:** Refinements Based on Think-Aloud Testing.

*Dental FearLess 1*.*0* Development Area Feedback	Changes made for *Dental FearLess 2*.*0*
Navigation	
View previously completed modules	Pause and rewind buttons added
Use progressive muscle relaxation and belly breathing anytime	Highlight guided skills with icons, make always accessible
App has no central place (“watching a movie”)	Home screen / menu developed
No sense of how much time is left in module	Progress bar added; new screens identify module subsections
Engagement	
Too much text during coaching voiceovers	Only key words appear during voiceovers
Needle exposure module too long	User can choose to proceed to next exposure despite fear rated as moderate
Too many question screens at beginning	Reduced number of questions, put key questions on a single screen and used sliders to answer
App too long	Progression through commonly used injection-fear module shortened
Lack of reward for progress through the app	Added common usability design elements (e.g., celebration of complete module)
Aesthetics	
"Textbook” appearance accompanying coaching screens is visually boring	Color-scheme changes; vastly reduced text

## Discussion

Overall results support the usability, feasibility, and acceptability of the *Dental FearLess 1*.*0* app. End-users gave fairly high overall ratings and positive reactions to the app’s general usability. In line with previous studies on the grading scale of SUS scores, our average rating amounted to a “good” or “B+” score for this beta version of the app [55,56]. *Dental FearLess* achieved a usability score significantly higher than the average (*M* = 68) found across a large-scale review of all mobile health apps [54], and similar to other freely available CBT-based mobile apps [57,58]. Moreover, end-users view the app-based treatment as mostly credible, meaning that its rationale was seen as logical and reasonable [51]; credibility ratings have been linked to symptom improvement in intervention research [52]. CEQ credibility ratings were slightly higher than those found in other recent studies of CBT-based mobile apps and were more similar to credibility ratings of in-person CBT treatment [59,60]. This may indicate that our attempts to make CBT-DF accessible via colloquial framing (e.g., what you think, feel, and do at the dentist; just test it) were successful, facilitating users’ perceptions of coherence. The app’s credibility ratings were bolstered by several participants remarking that they felt validated in their fear and empowered to ask for what they need at the dental office. In short, the app content and strategies were resonant or indicative of a “task-technology” fit for users [61]. Through think-aloud testing, we also identified several elements that needed to be altered based on end-users’ reports. In fact, some of the same feedback garnered during think-aloud testing was echoed via spontaneous comments made by participants who only completed the usability scales, further corroborating general feedback.

### Aesthetic and navigation

Through qualitative analysis of feedback, we uncovered two primary categories of design-related elements that threatened usability: aesthetics and navigation. Regarding aesthetics, users commented that although they loved the animations in the app, they found the interspersed coaching screens “boring” in comparison, with a “textbook”-like appearance and too much text per screen. Users also lamented the lack of navigability, commenting that most apps allow them more control and have a centralized screen, whereas this felt like an “interactive movie” they had to play through to the end. Some people commented that while they understood doing the entirety of the app in one sitting was ideal, it’s not the way they typically engage with health or fitness apps. Moreover, this structure prevented users from easily accessing the components of the app deemed most helpful.

Overall feedback on the lack of navigation implied that users want to feel they are actively choosing to engage in treatment when they want to and for how long they want to, rather than having it imposed on them during one lengthy session. In addition to adding navigation throughout, our solution to this was twofold. We attempted to accommodate users’ requests for control over the time spent on the app, making all modules available on demand (from the menu) after the initial and top-fear modules were completed. Furthermore, only one module at a time now must be completed during an app session.

Given the hundreds of thousands of sleeky produced, well-funded apps users have access to, criticisms regarding both aesthetics and navigation were unsurprising. In fact, a large-scale analysis of data from eight different m-health applications found interface design problems and navigation to be the second and fourth most common type of usability issues reported across testing [62].

### Fidelity and usability

Additionally, although the usability and credibility ratings would imply a notable degree of engagement, we also uncovered issues in app design that detracted from it. Notably, the choice to have a duplicative onscreen text of the spoken coach audio was distracting to participants. Similarly, users found the injection-fear module—adapted directly from the design of the evidence-based CARL injection program [24]—too lengthy. Although, in some ways, this implies habituation (in that users’ fears at the sight of the needle were reduced enough through the process for them to be bored), we worried about app discontinuation prior to the completion of the seven-step exposure hierarchy. Specifically, when participants rated a specific needle exposure segment as evoking any fear, they had to repeat that level before progressing. The strict stepped hierarchy program that necessitates significant reduction of fear at every stage is more representative of habituation, rather than an inhibitory learning model of exposure. Indeed, modern CBT-efficacy research indicates that the amount of fear reduction during exposure is not actually predictive of treatment success [40]. As such, we changed this requirement such that participants demonstrating mild-to-moderate fear were given the option of repeating that exposure or progressing to a more intense one.

Our change to the app injection module was evidence-informed and consistent with current theory regarding how exposure works mechanistically; these realities allowed us to be comfortable with the final version. However, the process of revising the exposure-based treatment (as opposed to an aesthetic / layout element) as originally designed is representative of a larger tradeoff implicit in m-health mobile app design: the negotiation of dosage to be sufficient versus the probability users will engage with the app and benefit from the treatment at all. A large-scale review of self-administered, CBT-based m-health apps for depression found that only a handful included comprehensive (i.e., included all key components of) CBT [63]. To maintain treatment fidelity, we ensured *Dental FearLess*’ comprehensive initial module devoted significant time to introducing each of the CBT-DF treatment components and set up a structure of (a) psychoeducation, (b) modeling, (c) and active practice that recurred throughout the app.

Each subsequent module of *Dental FearLess* includes all active ingredients of DF-CBT in some combination. Following testing, we made completion of the comprehensive first module and an individual’s highest-rated fear module (e.g., needles) mandatory for a user to unlock fast-forwarding capacities and access to additional modules. This decision was the result of our team’s attempt to negotiate a minimally sufficient treatment dosage with improved usability. However, only a randomized control trial can indicate whether any translation of an EBT to mobile-app makes for an *effective* treatment.

### Future directions

A related concern has centered around whether app-based psychological-treatments can be delivered in a way that appeals to users. After all, fidelity and effectiveness are rendered moot for an app nobody wants to use. Indeed, researchers and clinicians continue to ask a fundamental question regarding m-health apps: are they designed for engagement [64]? The resounding answer to which has been, not really. To increase engagement, CBT app developers have proposed modularization (such as we described above) and gamification (which we have incorporated in a very limited way) as ways to increase user engagement [65]. How to best accommodate end-users’ usability requirements while maintaining treatment fidelity is a fruitful and necessary area for future research. This may be especially relevant for CBT-based anxiety treatments because they necessitate exposure to objects of fear—an activity even competent therapists sometimes struggle to get their patients to participate in [66]. This is largely why *Dental FearLess* incorporates evidence-based strategies from other treatments [37–39] aimed at building motivation for, and increasing accessibility to, fear-recovery at the beginning of the app. Parsing out whether engagement is improved (i.e., more users stick with it) via the addition of such strategies to CBT apps for anxiety is another worthy direction for future research.

### Strengths and limitations

This study’s strengths included a mixed-methods and user-centered approach that allowed for comprehensive feedback regarding the usability and acceptability of *Dental FearLess 1*.*0*. Additionally, our sample size adhered to usability guidelines and, in recruiting from dental clinics and through referral practices, was representative of the target population [36]. Although most m-health interventions have similarly strong recruitment rates, many tend to have low completion rates as the accessibility and flexibility that m-health interventions provide have a counter-effect on program completion [46]. The typical low usage rate, however, did not apply because most m-health interventions are intended for prolonged use, whereas the *Dental FearLess* app caters towards usage at a specific time/context (e.g., the days leading up to a dental appointment, preparing to make an appointment).

This study also had several limitations. The app was tested at various points by fearful dental patients rather than at a standardized amount of time prior to a dental appointment (e.g., 1 day before), which its intended purpose is to prepare a user for. Therefore, we cannot know whether variations in time (and likely apprehension regarding the dental visit) may have impacted usability, credibility, or expectancy perceptions. Similarly, we do not have data on the number of participants who were screened and offered the chance to use the app, but declined, which could provide an alternative measure of overall acceptability among the intended population of fearful patients. Additionally, budgetary constraints limited the scale of our revisions. Furthermore, not all end-user suggestions could be addressed. particularly those for additional, expensive animations and for navigation in places that would allow skipping through specific module segments that are key CBT elements.

### Conclusion

Overall, the issues identified through our process of usability testing of a CBT-based m-health app—developed for use in a large, grant-funded RCT—echo those found across this literature: the nearly incompatible challenge of creating an app that simultaneously promotes user engagement and treatment fidelity. Furthermore, because avoidance is the primary maintainer of anxiety, we are exquisitely aware of how aversive pursuing treatment to combat one’s fears can be. It is no wonder users would want all the bells, whistles, and controls permissible to increase the tolerability of such a self-guided experience.

## Supporting information

S1 Data Basic Data. (XLSX)
